# GENDERED PATHWAY TO LIFE SATISFACTION VIA HELPING SPOUSES WITH DIGITAL TECHNOLOGY USE

**DOI:** 10.1093/geroni/igae098.2901

**Published:** 2024-12-31

**Authors:** Hayoung Park, Susanna Joo, Sun Ah Lee

**Affiliations:** Yonsei University, Seoul, Republic of Korea; Yonsei University, Seoul, Republic of Korea; The Pennsylvania State University, University Park, Pennsylvania, United States

## Abstract

Digital capabilities are closely related to the quality of life, and due to rapid digitalization, the exchange of help regarding digital technology use frequently occurs within close relationships in later life. As digital technology penetrates deeply into daily life, exploring the role of exchange of help regarding digital technology use within couple relationships could help specify daily interaction strategies improving the quality of life of individuals. This study explored how the husband and wife’s digital capabilities affect life satisfaction via helping their spouses with digital technology use within dyadic associations. For this, we used the Digital Aging in Dyads (DAID) data collected through an online survey in May 2023, targeting 257 older Korean straight couples aged 45 to 74. According to the modified Actor-Partner Interdependence Mediation Model analysis with bootstrapping, two mediating paths were found to be significant; the higher the digital capabilities of husbands, the more help with digital technology use provided by husbands to wives, which increases the life satisfaction of both husbands and wives. However, for wives, only the direct association between helping their husbands with digital technology use and wives’ life satisfaction was significant. These findings indicate gender asymmetric associations as the husband’s digital capabilities serve as greater power within later-life couple relationships whereas the wife’s digital capabilities nor helping their husbands with digital technology use do not play significant roles. This study is meaningful in presenting a new model for improving life satisfaction in the context of couple relationships in the digital era.

